# Answering research questions without calculating the mean

**DOI:** 10.3389/fpsyg.2015.01379

**Published:** 2015-09-08

**Authors:** Guillermo Campitelli

**Affiliations:** School of Psychology and Social Science, Edith Cowan UniversityPerth, WA, Australia

**Keywords:** research methods, mean, variability, deliberate practice, expertise

In an important theoretical article Speelman and McGann (2013) indicated that psychological researchers tend to use statistical procedures that involve calculating the mean of a variable in an uncritical manner. A typical procedure in psychological research consists of calculating the mean of some dependent variable in two or more samples and to present those means as summaries of the samples. The next step is to use some statistical technique (e.g., *t*-test, ANOVA) in order to be able to determine the probability of finding the observed differences between means in those samples given that the difference between the means of the populations from which the samples were extracted is zero. If this probability is very low (i.e., <0.05) the psychological researcher decides that the difference between the means of the populations of interest is not zero.

This procedure—the null hypothesis statistical significance testing (NHST) procedure–has received a huge number of criticisms, which are beyond the scope of this article. However, I would like to present the anecdote told by Cohen (1994), not only to criticize the NHST procedure itself but also the uncritical manner in which the procedure is used. Cohen tells us that a colleague hypothesized that a rare disease did not exist in a population; he then collected a sample of 30 individuals and found that one of them had the disease. He then wondered what type of significance test should be used in this situation. Obviously, the existence of one case with the disease is enough evidence to refute the hypothesis, but the uncritical search for a hypothesis testing procedure precluded the researcher from seeing the obvious.

This anecdote nicely dovetails with Speelman and McGann's (2013) assertion that psychological researchers tend to use procedures that involve calculating means in an uncritical manner. The goal of this article is to emphasize that there are procedures that do not involve calculating means, which are perfectly sound to answer research questions. In the following sections I will present the endeavor that other colleagues in the field of psychology of expertise and I embarked on with the purpose of testing hypotheses of the deliberate practice framework (Ericsson et al., 1993). I will present four measures that did not involve calculating the mean (i.e., variability, a value, a case, and distributions) I have used in my research to answer research questions. Before that I briefly explain the deliberate practice framework.

## Deliberate practice framework

Ericsson et al. (1993) presented the deliberate practice framework of expert performance. The framework provides recommendations of how to conduct research in the field of expertise, it defines what deliberate practice is and it states that abundant deliberate practice constitutes a *necessary* and a *sufficient* condition to achieve high levels of expertise (see Campitelli and Gobet, 2011; Ericsson, 2014; Hambrick et al., 2014a,b for a discussion about the hypotheses of the deliberate practice framework).

Ericsson et al. (1993) defined deliberate practice as engaging in highly structured domain-specific activities deliberately developed to correct technical mistakes and to improve performance, which are conducted with high concentration levels and are followed by immediate feedback (e.g., given by a coach). They indicated that these activities are not typically enjoyable and they distinguished deliberate practice from other activities such as work and play. The deliberate practice framework includes the strong statement that genetic differences among individuals do not explain differences in expert performance (except for the case of height in some sports such as basketball), and that genetic differences may only contribute to expert performance indirectly through deliberate practice (i.e., there may be genetic differences in the willingness to engage in long periods of deliberate practice).

As indicated by Campitelli and Gobet (2011) and Hambrick et al. (2014a) the deliberate practice framework claims that abundant deliberate practice is both a necessary and a sufficient condition to achieve high levels of expert performance in sports, games, arts, and science.

## Answering research questions with measures of variability

In a study conducted with 104 chess players (see Gobet and Campitelli, 2007; Campitelli and Gobet, 2008 for details), among other questions, Campitelli and Gobet requested participants to indicate the number of hours of individual and group practice they had engaged in since they started playing chess. The procedure was similar to the one used by previous researchers who mostly favor the deliberate practice framework (e.g., Charness et al., 1996, 2005).

In order to test the research question “Is abundant deliberate practice a *sufficient* condition to achieve high levels of expert performance in chess?” Campitelli and Gobet (2011) reviewed previous literature on chess expertise and utilized three procedures. In this section I focus on one of them: calculating the variability of the number of hours to achieve the master level—a level of expertise 3.5 standard deviations higher than the mean^1^. If the variability is small, this would give support to the deliberate practice framework whereas a large variability would provide evidence against that framework. This procedure was based on Gobet and Campitelli's (2007) dataset. Gobet and Campitelli had access to archival data that allowed them to determine the exact year in which the players achieved the master level. They used these data in combination with the number of hours of practice that each player accumulated until they achieved the master level. They then calculated the variability on the number of hours required to achieve that level. They found a range from 730 to 16,000 h of individual practice to achieve the master level. Thus, the deliberate practice framework's hypothesis that abundant deliberate practice is a sufficient condition to achieve high levels of expertise was not supported by the data.

## Answering research questions with one value

As indicated by Campitelli and Gobet (2011) another way of testing the above hypothesis is to find one individual who engaged in abundant deliberate practice and failed to attain the master level. This would rule out abundant deliberate practice as a sufficient condition to achieve high levels of expert performance. Campitelli and Gobet reported that there were several players dedicating more than 20,000 h to chess who did not achieve the master level; therefore, the hypothesis that deliberate practice is a sufficient condition was not supported by the data.

## Answering research questions with one case

Ericsson et al. (1993) hypothesized that 10 years of intense dedication to a field are *necessary* to achieve high levels of expert performance. This claim was slightly changed and popularized to the general public by the writer Malcom Gladwell in his bestseller “Outliers” (Gladwell, 2008). Appealing meritocratic values Gladwell captured the public imagination by coining the “10,000 h rule”: 10,000 h of intense dedication are *necessary* to achieve high levels of expert performance.

In order to test this hypothesis is not even necessary to collect data because archival data are available. Finding one case in which a high level of expert performance in chess is achieved in less than 10 years—in other words, finding a Mozart of chess–would refute the hypothesis. Indeed, Gobet and Campitelli (2007) identified more than one case: Ukranian Ruslan Ponomariov and Hungarian Peter Leko attained the grandmaster level (i.e., 2 levels [or 2 standard deviations] up the master level) at the age of 14, and in interviews they both reported having started playing chess at the age of 7. More impressively, Ukranian-born Russian Sergei Karjakin obtained the grandmaster level at the age of 12 and the international master level at the age of 11. At the age of 11 he was hired by Ponomariov to assist him in the preparation for the 2002 Chess World Championship match. More recently, the current world champion, Norwegian Magnus Carlsen obtained the grandmaster level at the age of 13 and reported that he played his first chess tournament at the age of 8 (see Gobet and Ereku, 2014, for more details on the case of Magnus Carlsen). Nowadays, there are 23 players who achieved the grandmaster level before the age of 15. These data suggest that 10 years or 10,000 h of intense dedication are not *necessary* to achieve high levels of expert performance.

## Answering research questions with distributions

Hambrick et al. (2014a) re-analyzed Gobet and Campitelli's (2007) data and presented a figure (see Figure 2, p. 39) with a distribution of hours of deliberate practice in three groups of chess players: master players, expert players and intermediate players (i.e., players with less than 2000 points ranging a number of categories). Although the mean hours of deliberate practice between groups differ [master *M* = 10,530 h (*SD* = 7414), expert *M* = 5673 h (*SD* = 4654), and intermediate *M* = 3179 h (*SD* = 4615)], as suggested by the large standard deviations, the overlap among the three distributions is evident by just visual inspection. For example, as expected, more than 60% of the intermediate players practiced between 0 and 2500 h. If abundant practice were a necessary condition to achieve high levels of expertise it is not expected to have players of the other groups in this interval of low practice. However, more than 25% of the expert players and more than 10% of the master players are in this interval. Moreover, the mode of the master and the expert groups is located in the same interval (i.e., between 5000 and 7500 h of practice), with more than 30% of experts, almost 25% of masters and almost 10% of intermediate players located in this interval. Furthermore, as expected, about 25% of the masters accumulated more than 17,500 h of deliberate practice; but, unexpectedly, about 2% of the experts and about 3% of the intermediates also accumulated more than 17,500 h of deliberate practice.

## Conclusion

As indicated by Speelman and McGann (2013), calculating a mean of some dependent variable as a first step of other statistical procedures is only one of a range of procedures available for the psychological researcher. There are two main reasons why psychological researchers tend to overlook the type of analyses presented above. First, psychological researchers are trained in application of statistical procedures that are typically useful for most types of research. Based on my experience with colleagues of other disciplines, this training is of high quality, thus psychological researchers have reasons to be proud of their analytic skills. However, the training focuses on the application, not the understanding, of those procedures. Indeed, research has shown that psychological researchers have difficulties in understanding *p* values (e.g., Badenes-Ribera et al., 2015). Second, psychology has a shortage of formal (i.e., mathematical, computational) theories that allow researchers to make precise numerical predictions of values (or a range of values) in experiments. This leads to relying on qualitative predictions (i.e., a group will have a higher average than another group) in which procedures involving calculating group means are the most appropriate. In this respect, Ericsson and colleagues should be credited for providing numerical predictions (i.e., 10 years (or 10,000 h) of deliberate practice are necessary to achieve high levels of expert performance), which can be tested with the analytic procedures explained above.

This article builds upon Speelman and McGann's (2013) call for critical use of statistical procedures, and illustrates four sound procedures to answer research questions, which do not involve calculating the mean. It is to be hoped that this article contributes toward the development of formal theories and ingenious procedures to answer research questions, as opposed to fitting research questions to the requirements of extant popular statistical procedures.

## Conflict of interest statement

The author declares that the research was conducted in the absence of any commercial or financial relationships that could be construed as a potential conflict of interest.

## Footnotes

^1^The chess international rating system uses the Elo (1978) scoring system, which follows a normal distribution with a theoretical mean of 1500 and standard deviation of 200, in which the current world champion possesses a score of 2876. The psychology of chess literature typically uses the following hierarchy to categorize chess players: >2600 = grandmaster, >2400 = international master, >2200 = Master, >2000 = Expert or candidate master, >1800 = Class A player, >1600 = Class B player, >1400 = Class C player.
